# Validation of the ^CSR^FENCE score for prediction of febrile neutropenia during chemotherapy cycles 2–6

**DOI:** 10.1007/s12672-022-00575-1

**Published:** 2022-10-17

**Authors:** Razan Zatarah, Nour Faqeer, Aseel Mahmoud, Tasnim Quraan, Lujain Matalka, Aya Kamal, Lama Nazer

**Affiliations:** Department of Pharmacy, King Hussein Cancer Center, Queen Rania Street, Al-Jubeiha, P.O. Box 1269, Amman, 11941 Jordan

**Keywords:** Chemotherapy-induced febrile neutropenia, Chemotherapy, Febrile neutropenia, Neoplasms, Prediction

## Abstract

**Purpose:**

Though febrile neutropenia (FN) risk prediction models are important in clinical practice, their external validation is limited. In this study, we validated the Cycle-Specific Risk of FEbrile Neutropenia after ChEmotherapy (^CSR^FENCE) score for predicting FN.

**Methods:**

We reviewed the medical records of patients with solid malignancies and diffuse large B-cell lymphoma during chemotherapy cycles 2–6 and recorded if patients developed FN, defined as absolute neutrophil counts less than 500 cells/microL with fever more than or equal to 38.2 ℃. The ^CSR^FENCE score was determined by adding the risk factors' coefficients described by the original study; subsequently, the score was used to classify chemotherapy cycles into the following risk groups for developing FN: low, intermediate, high, and very high risk. The discriminatory ability of the score was assessed using area under the receiver operating characteristics curve (AUROCC) and incidence rate ratios (IRR) within each ^CSR^FENCE risk group.

**Results:**

We analyzed 2870 chemotherapy cycles, of which 42 (1.5%) were associated with FN. Among those, 3 (7.1%), 14 (33.3%), 5 (12%), and 20 (47.6%) were classified as low, intermediate, high, and very high risk for developing FN, respectively. The AUROCC was 0.72 (95% CI 0.64–0.81). Compared with the low risk group (n = 666), the IRR of developing FN was 1.01 (95% CI 0.15–43.37), 0.69 (95% CI 0.08–32.46) and 1.17 (95% CI 0.17–49.49) in the intermediate (n = 1431), high (n = 498) and very high (n = 275) risk groups, respectively.

**Conclusion:**

The ^CSR^FENCE model can moderately stratify patients into four risk groups for predicting FN prior to chemotherapy cycles 2–6.

## Introduction

Febrile neutropenia (FN) is a life-threatening complication of myelosuppresive chemotherapy. FN may result in chemotherapy delays, reduced chemotherapy dose intensity and treatment discontinuation leading to suboptimal treatment effects of cancer, especially in patients receiving chemotherapy for curative intent [1]. Prophylactic use of granulocyte colony-stimulating factors (G-CSF) and antimicrobials, along with non-pharmacological measures, have been the cornerstones of FN prevention in patients at high risk for developing FN [2].

The guidelines recommend assessing the risk of FN at the start of each chemotherapy cycle to initiate preventive measures in high risk patients [3–5]. Current guidelines determine the overall risk of developing FN based on the anticipated risk associated with the administered chemotherapy regimen [3]. However, the chemotherapy regimen is one component of risk assessment and must be combined with patient specific risk factors and disease characteristics to estimate the individual’s overall risk of FN [3–5]. At present, there is no clear guidance in terms of how to determine the specific risk for FN based on the underlying risk factors. Thus, there is an increasing interest in developing risk prediction models to stratify patients based on their overall risk of developing FN before chemotherapy delivery, taking into consideration patient- and treatment- related factors.

The FEbrile Neutropenia after ChEmotherapy (FENCE) score is a clinical tool that was recently developed by Aagaard et al. to estimate the risk of developing FN at the first cycle of chemotherapy in treatment-naïve patients with solid tumors and diffuse large B-cell lymphoma (DLBCL) [6]. However, the FENCE score does not include several factors that may increase the risk of neutropenic fever in the following cycles, such as chemotherapy delay and previous episodes of FN. To address this issue, a cycle specific risk score was created to predict the risk of developing FN in cycles that follow the initial cycle of chemotherapy, specifically cycles 2 to 6, and referred to it as the “Cycle-Specific Risk of FEbrile Neutropenia after ChEmotherapy (^CSR^FENCE) score” [7]. Though Aagaard et al. suggested that the ^CSR^FENCE risk score had good discriminatory ability, there was no validation of that prediction score in external cohorts.

Therefore, we sought to externally validate the ^CSR^FENCE risk score in predicting FN prior to chemotherapy cycles 2–6 in a cohort of patients with solid malignancies and DLBCL.

## Methods

A retrospective chart review conducted between January 2019 and November 2019 at a comprehensive cancer center in Jordan, King Hussein Cancer Center (KHCC). The center is a 350-bed hospital that provides comprehensive cancer care to over 3500 new patients per year. The protocol was approved by the institutional review board of KHCC (study#20 KHCC188) with waiver of consent in view of the retrospective nature of the study [8]. The study was conducted in accordance with the Declaration of Helsinki and adhered to Good Clinical Practice guidelines.

Newly diagnosed adult patients with solid malignancies and DLBCL who had received their second cycle of chemotherapy were included in the study and their medical records were reviewed until discontinuation of chemotherapy, administration of a maximum of 6 chemotherapy cycles, death, or loss to follow-up, whichever came first. Based on the study by Aagaard et al., we excluded patients who received two alternating chemotherapy regimens, patients who received weekly platinum-based chemotherapy, patients who had undergone bone marrow transplantation and those who were receiving cancer-related treatment as part of an investigational study or compassionate protocol [6, 7].

We extracted the patients’ baseline characteristics, demographics, and the risk factors required for the ^CSR^FENCE score calculation, which included the FENCE risk group (based on pre-therapy risk factors used to calculate the FENCE score in the first cycle of chemotherapy), concurrent radiotherapy, cycle number, FN or neutropenia in previous cycles, and G-CSF prophylaxis [7, 8].

We followed the methodology to calculate the ^CSR^FENCE risk score for each patient’s individual cycle, as described in the original publication by Aagaard et al. First, we summed the assigned coefficients for each pre-therapy risk factor to calculate the ^CSR^FENCE score. Subsequently, we stratified each patient’s risk for developing FN prior to each chemotherapy cycle based on the calculated ^CSR^FENCE score as follows: low risk (score < 0), intermediate risk (score 1- 4), high risk (score 5—6), and very high risk (score ≥ 7) [7].

Patients were evaluated for any visits to the emergency department or admissions to the hospital that may have been associated with FN between cycles 2 and 6 of chemotherapy. We defined FN based on the criteria we used in our earlier study that evaluated the validity of the FENCE score and the criteria recommended by the clinical guidelines [8]. FN was defined as absolute neutrophil counts less than 500 cells/microL with fever more than or equal to 38.2 ℃ [3–5]. We recorded the outcomes following each FN episode including hospital admission, mortality, as well as chemotherapy dose reduction and dose delay in subsequent cycles.

### Statistical analysis

For descriptive statistics, median and interquartile range (IQR) were used to present continuous data, while numbers and percentages were used to present nominal data. The discriminatory ability of classifying patients into ^CSR^FENCE risk groups was determined by the area under the receiver operating characteristics curve (AUROCC) and incidence rate ratios (IRR), with their corresponding 95% confidence intervals (CI) within each ^CSR^FENCE risk group. In addition, a sensitivity analysis was performed to test the score performance when excluding patients who received G-CSF during their treatment. All analyses were performed with SAS version 9.4 (SAS Institute Inc, Cary, NC, USA) [8].

## Results

During the study period, 860 patients received a total of 2870 chemotherapy cycles, 2 through 6. The median follow-up was 3 cycles (IQR 2–4) with a median cycle length of 21 days (IQR 14–28). Table 1 outlines the characteristics of the patients prior to each chemotherapy cycle assessed.

**Table 1:** Baseline characteristics of the included patients at the time of each chemotherapy cycle (n = 2870 cycles)

Characteristics	FN N = 42	No FN N = 2828
Gender		
Male	18 (42.9%)	1024 (36.2%)
Female	24 (57.1%)	1804 (63.8%)
Age, years		
Median (IQR)	55 (44–63)	55 (45–63)
Cancer type		
Breast	20 (47.6%)	1191 (42.1%)
DLBCL	10 (23.8%)	144 (5.1%)
Prostate	4 (9.5%)	50 (1.8%)
Non- small cell lung	2 (4.7%)	155 (5.5%)
Small cell lung	1 (2.4%)	65 (2.3%)
Colorectal	1 (2.4%)	604 (21.4%)
Gastric	1 (2.4%)	162 (5.7%)
Cervical/endometrial	0	140 (4.9%)
Bladder	0	51 (1.8%)
Head and neck	0	28 (1%)
Others	3 (7.2%)	238 (8.4%)
Disease Stage		
Adjuvant/Ann Arbor I	7 (16.7%)	631 (22.3%)
Neoadjuvant or concomitant/ Ann Arbor II	15 (35.7%)	1024 (36.2%)
Locally advanced or disseminated/ Ann Arbor III +	20 (47.6%)	1173 (41.5%)
FENCE risk group^b^		
Low (score ≤ 16)	10 (23.8%)	1041 (36.8%)
Intermediate (score 17 -35)	13 (31.0%)	844 (29.8%)
High (score 36 – 52)	6 (14.3%)	513 (18.1%)
Very high (score ≥ 53)	13 (31.0%)	430 (15.2%)
Platinums		
Yes	7 (16.7%)	1373 (48.6%)
Taxanes		
Yes	8 (19.0%)	470 (16.6%)
Concurrent radiotherapy^a^		
Yes	3 (7.1%)	120 (4.2%)
Cycle number		
2	14 (33.3%)	845 (29.9%)
3	12 (28.6%)	785 (27.8%)
4	8 (19.0%)	655 (23.2%)
5	3 (7.1%)	289 (10.2%)
6	5 (11.9%)	254 (9.0%)
FN or neutropenia in previous cycle		
No neutropenia	14 (33.3%)	2315 (81.9%)
Neutropenia, but not FN	4 (9.5%)	364 (12.9%)
1 FN event	19 (45.2%)	131 (4.6%)
> 1 FN event	5 (11.9%)	18 (0.6%)
G-CSF primary prophylaxis		
Yes	12 (28.6%)	273 (9.7%)
Antibiotic prophylaxis		
Yes	0	26 (0.9%)
^CSR^FENCE risk group		
Low (score ≤ 0)	3 (7.1%)	663 (23.4%)
Intermediate (score 1–4)	14 (33.3%)	1417 (50.1%)
High (score 5–6)	5 (11.9%)	493 (17.4%)
Very high (score ≥ 7)	20 (47.6%)	255 (9.0%)

The column percentages represent the proportion of cycles based on the total number of cycles that were associated and those that were not associated with FN in each category

*IQR* Interquartile range, *FN* febrile neutropenia, *DLBCL* diffuse large B-cell lymphoma, *G-CSF* granulocyte colony-stimulating factors

^a^Radiotherapy: concurrent radiotherapy during cycle

^b^FENCE risk group: calculated based on pre-therapy risk factors including: sex, age, cancer type, disease stage, albumin, bilirubin, estimated glomerular filtration rate, infection before chemotherapy, number of and type of chemotherapy drugs [6]

FN was reported in 42 (1.5%) cycles in which one third of the episodes occurred after cycle two (n = 14, 33.3%) and about one third occurred after cycle three (n = 12, 28.6%). Among the reported FN episodes, the majority required hospitalization (n = 35, 83.3%) while the remaining were managed in the emergency department. All patients were discharged from the hospital. Dose reductions and chemotherapy delays occurred in 6 (14.3%) and 9 (21.4%) of the subsequent cycles, respectively.

**Fig. 1:**
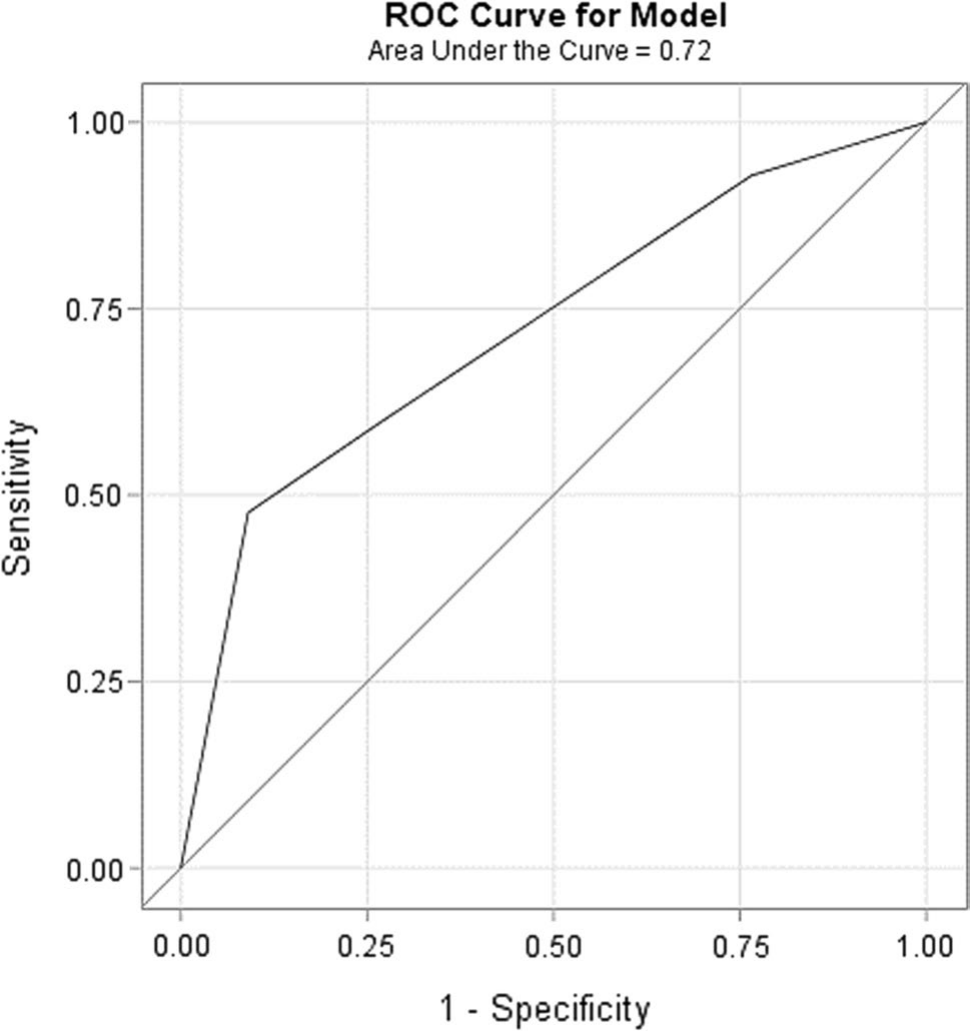
Receiver operating characteristic (ROC) curve for prediction of febrile neutropenia based on ^CSR^FENCE risk groups. Area under the ROC curve (AUROCC) = 0.72 (95% CI 0.64–0.81)

According to the ^CSR^FENCE risk group classification, the chemotherapy cycles administered were considered as being low risk (n = 666, 23.2%), intermediate (n = 1431, 49.8%), high (n = 498, 17.4%) and very high (n = 275, 9.6%) risk for FN. Among the reported FN episodes, 3 (7.1%) were reported in the low risk, 14 (33.3%) were reported in the intermediate, 5 (12%) were reported in the high risk, and 20 (47.6%) were reported in the very high risk group. The AUROCC was 0.72 (95% CI 0.64– 0.81) (Fig. 1). When excluding patients who were on G-CSF, sensitivity analysis resulted in an AUROCC of 0.72 (95% CI 0.62–0.82).

The IRR per point increase in ^CSR^FENCE score was 2.62 (95% CI 1.80–3.82). Compared to those at low risk, the IRR of developing FN was 1.01 (95% CI 0.15–34.37), 0.69 (95% CI 0.08 – 32.46) and 1.17 (95% CI 0.17–49.49) in the intermediate, high, and very high risk groups, respectively.

## Discussion

In this study, we aimed to assess the ability of utilizing the ^CSR^FENCE risk groups in predicting the risk of developing FN during chemotherapy cycles 2–6 in an independent dataset. When applied to our cohort, the classification based on the ^CSR^FENCE risk groups demonstrated a moderate discriminatory ability for predicting FN. This was consistent with the results of the validation cohort of the original study by Aagaard et al., who also reported good discriminatory ability to predict the underlying risk of FN at chemotherapy cycle initiation and concluded that a model utilizing cycle-specific risk factors performs better than one that solely uses pre-therapy data [7].

Although the FENCE score was used to predict the risk of FN in solid tumors and DLBCL during first cycle of chemotherapy, Aagaard et al. were unable to track treatment changes in DLBCL patients in subsequent chemotherapy cycles and therefore did not include them in the ^CSR^FENCE score. However, in our study, we decided to include DLBCL patients within our validation cohort since the FENCE risk group, which included DLBCL patients, is one of the items used to calculate the ^CSR^FENCE score.

The population in the current study were younger at baseline (median 55 years IQR 44–63) compared to the original study (median 64 years IQR 54–71), where notably age > 65 is considered a risk factor for developing FN [3]. The majority of our patients were initially classified in the low (36.6%) and intermediate (29.9%) FENCE risk groups for developing FN after cycle one of chemotherapy. Furthermore, our population was predominately breast cancer patients (42%), who also made up the majority of FN cases (47%). This may impact the generalizability of our findings to other types of cancer. In addition, about 80% of our patients did not experience previous neutropenia or FN in any of their cycles, and as suggested by the guidelines, having previous episode of FN or a dose-limiting neutropenic event increases the overall FN risk to a high risk group [3].

Our findings showed a significant 2.6-fold increase in the incidence of FN per point increase in the ^CSR^FENCE score. However, the differences in IRR between the stratified risk groups were not statistically significant, and the confidence intervals were wide. This could be explained by the relatively few reported FN events (1.5%) and possibly a limited power to detect a significant difference between the ^CSR^FENCE risk groups.

The incidence of FN (1.5%) reported in our study was relatively similar to that reported by Aagaard et al. (2.1%). In our study, we used the narrow definition of FN, which is utilized by our center and more commonly used in clinical practice [3–5]. Due to the lack of certain data in the patients’ records in the original study such as temperature, Aagaard et al. used a wide definition for FN, which included any blood culture or death within 3 days of a neutrophil count < 0.5 × 10^9^/L or a leukocyte count < 2 × 10^9^/L. However, they reported in their sensitivity analysis that the discriminatory ability of the score was similar between the wide and narrow guideline definition of FN (i.e. documented fever and neutropenia) [6, 7].

In this study, the majority of the patients experienced FN following cycles 2 and 3, compared to subsequent cycles. A prospective study by Culakova et al. that evaluated the time course of neutropenic events in patients with early-stage breast cancer concluded that the risk of FN was greatest in the first cycle when most patients receive full-dose chemotherapy. Moreover, they reported a decrease in the incidence of FN during subsequent cycles due to reduced dose intensity of chemotherapy or increased use of supportive care measures [9]. In the present study, we cannot draw a clear conclusion regarding the effect of changes in dose intensity in the subsequent cycles due to infrequent occurrence of FN in our cohort.

Although the main aim of developing the ^CSR^FENCE score was to assess risk factors that only appear in later cycles such as chemotherapy dose delay and reduction, Aagaard et al. did not include them as risk factors when determining the ^CSR^FENCE risk groups. They assumed that there was a high level of correlation between these risk factors and prior FN or neutropenia which were identified as strong predictors of future FN in the subsequent cycles and were included in the final score.

Few studies have addressed the cycle-specific risk factors for predicting FN beyond the first cycle of chemotherapy. Among those, some have assumed that the risk of FN in cycle 1 can be extrapolated to subsequent cycles [10], while others have focused on a single cancer type such as breast cancer [11] or specific risk factors such as early lymphopenia [12]. Furthermore, limited accuracy of some prediction models were reported [11, 13]. Given these limitations as well as the heterogeneity of the published data, it is difficult to compare between the studies [14].

To our knowledge, this represents the first study to validate the ^CSR^FENCE risk groups in an external population. In addition, we included a relatively large cohort of patients with variable risk factors and chemotherapy regimens which would help in the understanding of the performance of the score in different cohorts before clinical implementation.

The current study has several limitations. First, it was conducted at a single center that might not be generalizable to other cancer centers with different healthcare practices. Secondly, patient's data were collected through a retrospective chart review and were limited by the quality of data documented. Third, as discussed in our recent publication, we did not look into independent risk factors for FN such as the dose intensity of the administered chemotherapy, co-morbidities, bone marrow involvement, corticosteroids and other immunosuppressants, as well as the performance status, which would add to the existing literature and might reveal important risk factors [8].

The ^CSR^FENCE score showed moderate discriminatory ability for identifying patients who are at high risk of developing FN during chemotherapy cycles 2–6 and hence could guide physicians in tailoring patient's treatment goals and initiate preventive measures based on pre-defined risk assessment. This may reduce the complications of FN and maintain the chemotherapy dose intensity. However, our findings indicate that the ^CSR^FENCE risk score necessitates some refinements before being used in clinical practice.

## Conclusion

Our study demonstrated that the ^CSR^FENCE model can moderately stratify patients into four risk groups for predicting FN prior to chemotherapy cycles 2–6. Further prospective validation addressing the ^CSR^FENCE risk score limitations across diverse cohorts are needed before clinical adoption.

## Data Availability

The data used in this study are available from the corresponding author, upon request.

## Abbreviations

AUROCC: Area under the receiver operating characteristics curve
CI: Confidence interval
^CSR^FENCE: Cycle-Specific Risk of FEbrile Neutropenia after ChEmotherapy
DLBCL: Diffuse large B-cell lymphoma
FENCE: FEbrile Neutropenia after ChEmotherapy
FN: Febrile neutropenia
G-CSF: Granulocyte colony-stimulating factors
IQR: Interquartile range
IRR: Incidence rate ratio
KHCC: King Hussein Cancer Center
ROC: Receiver operating characteristic
